# Altered Reward Processing and Sex Differences in Chronic Pain

**DOI:** 10.3389/fnins.2022.889849

**Published:** 2022-06-07

**Authors:** Anne K. Baker, Lauren C. Ericksen, Vincent Koppelmans, Brian J. Mickey, Katherine T. Martucci, Jon-Kar Zubieta, Tiffany M. Love

**Affiliations:** ^1^Department of Psychiatry, Huntsman Mental Health Institute, University of Utah, Salt Lake City, UT, United States; ^2^Department of Anesthesiology, Duke University School of Medicine, Durham, NC, United States; ^3^Center for Translational Pain Medicine, Duke University Medical Center, Durham, NC, United States; ^4^Department of Psychiatry, University of Michigan, Ann Arbor, MI, United States; ^5^Department of Psychiatry, Northwell Health, John T. Mather Memorial Hospital, Port Jefferson, NY, United States

**Keywords:** chronic pain, sex differences, reward processing, fMRI, striatum

## Abstract

Chronic pain and reward processing are understood to be reciprocally related to one another. Previous studies of reward processing in chronic pain patients have reported incongruent findings. While several factors likely contribute to these disparate findings, these previous studies did not stratify their analyses by sex—a factor previously shown to robustly impact reward-related responses. Thus, we examined sex as a factor of interest in level of striatal activation during anticipation of monetary incentives among patients with chronic non-specific back pain and healthy controls (HC). This study utilized functional magnetic resonance imaging during a monetary incentive delay task to evaluate reward and loss responsivity in the striatum among males and females with and without chronic pain (*N* = 90). Group, sex, and group-by-sex interactions were analyzed *via* repeated measures analysis of variance. Among HC, males exhibited significantly greater blood oxygen level dependent (BOLD) signal in the striatum during reward anticipation, particularly during large reward trials. By contrast, no significant sex differences were observed among patients. A significant group-by-sex interaction was also observed, revealing diminished BOLD responses among males with chronic pain relative to control males. These results provide novel evidence of sex-specific reductions in anticipatory responses to reward in patients with chronic pain. Altered striatal reward responsivity among males, but not females, suggests that the reward systems of males and females are uniquely disrupted by chronic pain, and highlights the value of including sex as a factor of interest in future studies of reward responsivity in the context of persistent pain.

## Introduction

Chronic pain is an exceedingly common health condition impacting 1 in every 5 adults in the United States (Yong et al., 2022). Sex-specific differences in the prevalence, severity, and clinical presentation of chronic pain are well documented (Mogil, 2020; Yong et al., 2022); however, the neurobiological origins of such sex differences in chronic pain and the mechanisms that more broadly lead to the development of these conditions are still not well understood. Increasing evidence suggests neuroadaptations within reward circuits may contribute to the pathophysiology of chronic pain (Elvemo et al., 2015; Finan et al., 2018).

Chronic pain, emotional processing and responding, and reward processing are regulated by overlapping brain regions, including core regions within the mesolimbic dopamine pathway like the ventral striatum (Becker et al., 2012; Navratilova and Porreca, 2014). Activity within the ventral striatum has long been known to play an integral role in motivated behavior and designation of salience (Salamone and Correa, 2012). While noted for its involvement in reward, the ventral striatum is also responsive to pain, stressors, and negative emotional states (e.g., Thierry et al., 1976; Scott et al., 2006). For example, both acute pain and pain relief have been shown to enhance mesolimbic activity in humans (Scott et al., 2006; Navratilova et al., 2012; Becerra et al., 2013). In the context of chronic pain, some have speculated (Borsook et al., 2016) that protracted recruitment of the mesolimbic dopamine system by pain may induce changes within regions like the ventral striatum, leading to blunted reward- and stress- responsivity and exacerbation of pain states. Indeed, reductions in striatal dopamine D2 receptor activity have been associated with enhanced pain responses and the development of persistent pain in animal models (Morgan and Franklin, 1991; Chang et al., 2014; Schwartz et al., 2014).

While studies on this topic in humans are still limited, the current body of research suggests persons with chronic pain may exhibit dysregulated reward-related activity (Baliki et al., 2010; Martikainen et al., 2013, 2015; Martucci et al., 2018; Kim et al., 2020; Yu et al., 2020); however, the results from these studies have been inconsistent. For example, using the well-established monetary incentive delay (MID) task (Knutson et al., 2000) that was designed to assess neural responses to anticipation of reward, Kim et al. (2020) reported significantly blunted striatal activation among a mixed sample of male and female patients with chronic low back pain and fibromyalgia, relative to healthy controls (HC). By contrast, in a study of females with fibromyalgia, Martucci et al. (2018) found no significant differences between patients and controls in blood oxygen level dependent (BOLD) signal in the ventral striatum during anticipation of reward. The source of such divergent findings is unclear. However, given that sex is a highly relevant factor in the context of reward, it is possible that differences in the make-up of participant cohorts in previous studies may partially explain these inconsistent findings.

Sex differences in the reward system have been repeatedly observed previously. In humans, positron emission tomography research has shown dopaminergic activity in the ventral striatum following amphetamine (Munro et al., 2006) and nicotine (Cosgrove et al., 2014) exposure was greater among males relative to females. More recently, Warthen et al. (2020) demonstrated greater activation of the striatum among males, along with behavioral sex differences, during anticipation of monetary rewards and losses. Taken together, previously observed sex differences in reward processing and chronic pain suggest it possible if not likely that sex may differentially influence neurobiological substrates of chronic pain and concomitant affective and reward-related processes. In order to fully understand these relationships, it is essential to examine the extent to which sexual dimorphisms may influence reward processing in the context of chronic pain. Consideration of sex differences will bolster our understanding of what contributes to the development and maintenance of chronic pain.

The present study aims to build upon and clarify mixed findings of ventral striatal activity during reward anticipation from Martucci et al. (2018) and Kim et al. (2020) by examining sex as a factor of interest. To accomplish this, we utilized fMRI during a MID task to examine the effects of group, sex, and group by sex interactions on BOLD signal during reward anticipation in a sample of non-specific back pain (CNBP) patients and HC. We hypothesized that (1) relative to controls, CNBP patients would exhibit significantly lower striatal activation during anticipation of reward, and (2) this effect would be sex specific.

## Materials and Methods

### Participants

This study included participants recruited from the University of Michigan as part of two parallel studies utilizing the MID task to explore reward-related processing in HC and chronic pain patients. A total of 97 participants successfully completed the MID task. Four subjects were excluded for excessive motion [> 20% volumes exceeded the 0.5 mm framewise displacement (FD) threshold] and 3 subjects had other functional data abnormalities; accordingly, 90 participants were left for analysis: 50 HC participants (30 female, 20 male, mean age ± SD = 33 ± 10.4) and 40 CNBP patients (22 female, 18 male, mean age ± SD = 38 ± 9.3). Sex was self-reported as that assigned at birth and was not distinguished from gender.

All participants provided written informed consent prior to study procedures, and all study protocols were approved by the University of Michigan’s Institutional Review Board. All participants were right-handed adults. HC had no history of psychiatric disorders and were recruited *via* advertisement. Patients were recruited from a local pain clinic and had a history of at least 1 year of chronic, non-specific back or neck pain; these participants self-reported pain levels of 3–8 on a 0–10 verbal rating scale. Additionally, CNBP participants did not endorse lifetime substance dependence, current nicotine dependence, or regular use of medications that had known central nervous system activity.

### Study Procedures

#### Monetary Incentive Delay Task

The MID task was selected for use during fMRI as way to assess neural representations of reward processing. When examining neural activity during anticipation of reward and loss, this task is known to robustly recruit the ventral striatum (Knutson et al., 2001; Cooper and Knutson, 2008). The MID task has been described in considerable detail previously (see Bartley and Fillingim, 2013; Mickey et al., 2016; Martucci et al., 2018 for enhanced description and task visualization); in brief, this task requires participants to respond *via* button press to a target in order to win or avoid losing money. During each 6 s trial, participants were presented with a series of cues indicating which type of incentive was at stake. This was followed by a fixation cross (2,500 ms), during which individuals anticipated being able to work for a reward or avoid a loss. Then, participants were presented with a response target, to which they were instructed to respond as fast and accurately as possible in order to successfully win or avoid losing money. Immediately following the target, participants received feedback on their performance. The task was adjusted to ensure approximately 66% accuracy.

The MID task was presented using Eprime (Psychology Software Tools, Sharpsburg, PA, United States). All subjects participated in two consecutive MID runs, each consisting of 70 pseudo-randomized conditions from the following condition types: large, medium, and small rewards (+$5.00 +$1.00, +$0.20, respectively), large, medium, and small losses (−$5.00, −$1.00, −$0.20, respectively), and neutral (±$0).

#### Image Acquisition and Preprocessing

Whole brain functional images were acquired on one of two General Electric scanners: a 3T Signa Excite II System or a 3T Discovery MR750 System. T2*-weighted images were collected (29 axial slices parallel to the AC-PC line, TR = 2,000 ms, TE = 30 ms, flip angle 90°, slice thickness = 4 mm, field of view = 22 cm, 64 × 64 matrix). Two sessions of 210 volumes were acquired and analyzed. Structural images were acquired for anatomic normalization using T1-weighted, 3D inversion recovery-prepared fast spoiled gradient recalled sequences (see Supplementary Materials and Methods).

All neuroimaging data were preprocessed using fMRIPrep (Poldrack Lab, Stanford, CA, United States) version 1.3.0.post3 (Esteban et al., 2019). Data were corrected for slice-timing and motion, then co-registered and normalized to Montreal Neurological Institute (MNI) standard space. Subsequently, the preprocessed functional images were smoothed with a 6 mm Gaussian kernel in SPM12 (Statistical Parametric Mapping, Functional Imaging Laboratory, London, England). See Supplementary Materials and Methods for further information.

#### First Level Models

An individual task model for each participant was constructed using a general linear model within SPM12. First level models included parameters of interest corresponding to the anticipation of each condition type, modeled as impulse functions following presentation of the 2,500 ms fixation cross and six head motion parameters (3 translations, 3 rotations). Volumes with excessive motions (FD > 0.5 mm) were included as confounds in the model (Siegel et al., 2014). To account for temporal autocorrelation, the FAST autocorrelation algorithm within SPM12 was used. An explicit brain mask generated by fMRIPrep 1.3.0.post3 was also applied (Olszowy et al., 2019). Parameter estimates for each contrast of interest (large, medium, small rewards and large, medium, small losses—all relative to neutral) were computed to characterize the hemodynamic response during the anticipatory phase of the task.

#### Region of Interest Analyses

We used an *a priori* region of interest (ROI) approach to explore differences in BOLD signal among CNBP patients and HC. We focused on the ventral striatum due to previously discussed divergent results (Martucci et al., 2018; Kim et al., 2020) within this region and, more broadly, due to its relevance in pain, affect and incentive salience, and corresponding functionality during the MID task. ROIs were created by placing 5 mm diameter spheres centered on ventral striatal coordinates reported by a recent ALE meta-analysis of 50 MID studies (*x, y, z:* left, −12, −8, −4; right, −12, 6, 0) (Oldham et al., 2018). We calculated the mean BOLD contrast estimate across all voxels in the left and right ROIs, representing average ventral striatal activity.

#### Questionnaires

Emotional states were measured among a subset of individuals immediately prior to the fMRI scan. State affect was measured using the Positive and Negative Affect Schedule (PANAS) (Watson et al., 1988)—a 60-item instrument designed to capture transient affect. Negative mood was probed using the composite Total Mood Disturbance (TMD) score from the Profile of Mood States (POMS) (McNair et al., 1971), and subjective state anxiety was assessed using the 20-item state scale from the Spielberger State-Trait Anxiety Inventory (STAI) (Spielberger, 1983). Among CNBP patients, the McGill Pain Questionnaire (Melzack, 1975) was collected as an integrative measure of back pain to assess the quality and intensity of their pain.

#### Statistical Analysis

All statistical analyses were conducted using IBM SPSS 26 (IBM Corporation, Armonk, NY, United States). Comparisons between groups were performed using *t*-tests, univariate analysis of variance, and repeated measures analysis of variance. Associations between BOLD signal and psychophysical measures were computed with two-tailed Pearson correlations, calculated separately for males and females.

## Results

### General Characteristics

Baseline characteristics of CNBP patients and corresponding HC participants are shown in Table 1. Age and differences in emotional states were evaluated using two-way analysis of variance (ANOVA) with group and sex as factors of interest. For age, the overall model trended toward, but did not reach, statistical significance [*F*_(1_,_87)_ = 2.6, *p* = 0.06]. See Table 1 for age comparisons by group, sex, and group-by-sex.

**TABLE 1:** Baseline characteristics.

	Control		Chronic pain		Analyses					
	Male	Female	Male	Female	Sex		Group		Group × Sex	
	Mean (SD)	Mean (SD)	Mean (SD)	Mean (SD)	F	*p*	F	p	F	p
Age	30.75 (9.89)	33.77 (10.67)	38.65 (7.88)	37.48 (10.42)	0.19	0.67	7.33	< 0.01	0.96	0.33
**Affect**										
PANAS negative**	10.75 (1.04)	11.27 (2.37)	15.41 (6.35)	12.04 (3.17)	1.72	0.2	6.24	**0.02**	3.18	0.08
PANAS positive**	29.63 (4.10)	33.2 (9.56)	29.65 (6.9)	24.83 (8.09)	0.09	0.77	3.89	0.05	3.93	**0.05**
POMS TMD**	-3.25 (5.75)	-2.07 (10.94)	21.71 (23.88)	10.67 (12.94)	1.29	0.26	18.88	**< 0.01**	1.99	0.16
STAI State Anxiety**	29.63 (7.96)	29.20 (6.21)	40.94 (12.16)	37.19 (9.76)	0.63	0.43	13.54	**< 0.01**	0.40	0.53
**Pain**										
McGill pain intensity	–	–	45.0 (21.14)	58.09 (21.87)	1.90*	0.07*	–	–	–	–
McGill pain unpleasantness**	–	–	46.29 (23.38)	57.73 (21.53)	1.6*	0.12*	–	–	–	–

Analyses represent the results of a two-way ANOVA with Sex and Group as factors except where denoted by (*), which indicates use of a two-sample t-test. PANAS, Positive and Negative Affect Schedule, POMS TMD, Profile of Mood States Total Mood Disturbance, STAI, State-Trait Anxiety Inventory. Double asterisks indicate analyses with reduced sample sizes as follows: PANAS Negative, n = 63; PANAS Positive, n = 63; POMS TMD, n = 61; STAI State Anxiety, n = 60; McGill Pain Unpleasantness, n = 39. Affect denotes affective measures. Pain denotes pain measures.

There was a significant difference in mean PANAS negative affect scores [*F*_(1_,_59)_ = 4.04, *p* = 0.11]; specifically we observed a significant main effect of group [*F*_(1_,_59)_ = 6.24, *p* = 0.02], with patients endorsing higher levels of negative affect than controls. Similarly mean differences were noted in PANAS positive affect [*F*_(1_,_59)_ = 3.67, *p* = 0.02], with a significant group-by-sex interaction [*F*_(1_,_59)_ = 3.93, *p* = 0.05] showing female controls endorsed the highest levels of positive affect and female patients the lowest, while male patients and controls endorsed nearly identical levels of positive affect. We also observed significant differences in POMS Total Mood Disturbance (POMS-TMD) scores [*F*_(1_,_57)_ = 7.71, *p* < 0.001], with a significant main effect of group [*F*_(1_,_59)_ = 18.88, *p* < 0.001], demonstrating patients reported significantly greater levels of mood disturbance than controls. Finally, we also noted significant differences in state anxiety [STAI; *F*_(1_,_56)_ = 5.11, *p* = 0.003], with a significant main effect of group [*F*_(1_,_56)_ = 13.54, *p* = 0.001] indicating greater rates of state anxiety among patients relative to controls.

Two-sample tests between patient males and females did not reveal any differences in clinical back pain intensity as measured by the McGill Pain Questionnaire [*t*_(38)_ = 1.90, *p* = 0.07], but females trended toward greater pain intensity than males. Female patients also endorsed higher levels of current back pain unpleasantness, though these ratings were again not significantly different from males [*t*_(37)_ = 1.6, *p* = 0.12].

### Region of Interest Activation: Ventral Striatum

To assess the effects of group and sex on average ventral striatal BOLD activation during reward and loss anticipation, we conducted a repeated-measures ANOVA. Incentive valence (reward, loss) and salience (large, medium, small) were included as within-subject factors, sex and group were entered as between-subject factors, and age was entered as a covariate of no interest.

**FIGURE 1:**
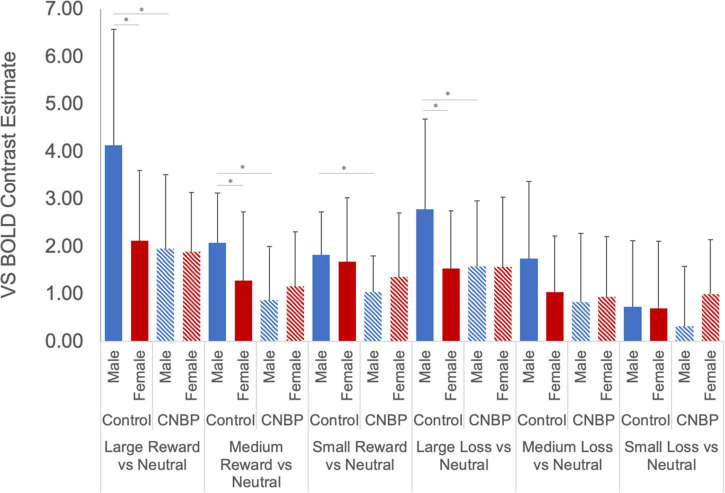
Group by sex *interactions during reward and loss anticipation.* During large reward trials (relative to neutral), control males exhibited significantly greater reward-related activation than control females; this difference was not observed between patient males and females. Significant differences were observed between control males and females for large rewards (*p* = 0.002) and medium rewards (*p* = 0.04). No such differences were observed between patient males and females (*p* = 0.89, *p* = 0.42, respectively). Significant differences were also observed between control males and patient males for large rewards (*p* = 0.002), medium rewards (*p* = 0.001), and small rewards (*p* = 0.005). No such differences were noted between control females and patient females (*p* > 0.40). During large loss trials (relative to neutral), control males exhibited significantly greater loss-related activation than control females (*p* = 0.02); this difference was not observed between patient males and females. Significant differences were also observed between control males and patient males for large rewards (*p* = 0.04). No such differences were noted between control females and patient females. Error bars represent confidence intervals. VS, ventral striatum. * denotes significant differences.

Results showed a novel, significant group-by-sex interaction effect [*F*_(1_,_85)_ = 3.99, *p* = 0.049; see Figure 1 and Table 2]. *Post hoc* tests revealed control males, relative to control females, exhibited greater ventral striatal BOLD activation during anticipation of rewards (*p* = 0.01) and avoidance of loss (*p* = 0.05); these results are consistent with previously observed reward processing sex differences among HC. On the other hand, no such differences were observed between patient males and patient females (All Reward vs. Neutral, *p* = 0.58; All Loss vs. Neutral, *p* = 0.70), essentially representing a negation of normally apparent sex-differences in reward processing. Relative to control males, patient males showed significantly less ventral striatal activation across all incentives (*p* = 0.007), while activation was similar among patient and control females (*p* = 0.80).

**TABLE 2:** Within- and between-group differences in MID activity.

	Chronic Pain				Control				Male		Female	
	Male	Female			Male	Female			Patient vs. control		Patient vs. control	
						
	(*n* = 17)	(*n* = 23)			(*n* = 20)	(*n* = 30)						
	Mean (SD)	Mean (SD)	*t*	*p*	Mean (SD)	Mean (SD)	*t*	*p*	*t*	*p*	*t*	*p*
**Reward**												
All vs. Neutral	1.28 (1.02)	1.47 (1.03)	0.6	0.58	2.68 (1.30)	1.69 (1.25)	2.7	**0.01**	3.6	**0.001**	0.7	0.49
Large vs. Neutral	1.95 (1.56)	1.88 (1.25)	0.1	0.89	4.13 (2.44)	2.12 (1.48)	3.3	**0.002**	3.2	**0.003**	0.6	0.54
Medium vs. Neutral	0.87 (1.13)	1.16 (1.13)	0.8	0.42	2.07 (1.05)	1.28 (1.44)	2.1	**0.04**	3.3	**0.002**	0.3	0.76
Small vs. Neutral	1.03 (0.77)	1.35 (1.35)	0.9	0.39	1.82 (0.90)	1.67 (1.35)	0.4	0.66	2.9	**0.007**	0.9	0.40
**Loss**												
All vs. Neutral	0.91 (1.21)	1.16 (1.12)	0.7	0.5	1.75 (1.25)	1.08 (1.07)	2.0	**0.05**	2.1	**0.05**	0.2	0.80
Large vs. Neutral	1.58 (1.38)	1.56 (1.46)	0.03	0.98	2.78 (1.90)	1.53 (1.22)	2.6	**0.02**	2.2	**0.04**	0.09	0.93
Medium vs. Neutral	0.82 (1.44)	0.92 (1.27)	0.25	0.81	1.74 (1.62)	1.03 (1.19)	1.8	0.08	1.8	0.08	0.3	0.75
Small vs. Neutral	0.32 (1.25)	0.99 (1.15)	1.7	0.09	0.73 (1.39)	0.69 (1.42)	0.1	0.92	0.9	0.36	0.8	0.41
**All incentives**												
All vs. Neutral	1.09 (1.10)	1.31 (0.98)	0.7	0.51	2.21 (1.23)	1.39 (1.09)	2.5	**0.02**	2.9	**0.007**	0.3	0.8
Large vs. Neutral	1.76 (1.38)	1.72 (1.22)	0.09	0.93	3.46 (2.09)	1.83 (1.25)	3.1	**0.004**	2.8	**0.007**	0.3	0.77
Medium vs. Neutral	0.84 (1.21)	1.05 (1.08)	0.5	0.59	1.91 (1.19)	1.16 (1.18)	2.2	**0.03**	2.7	**0.01**	0.4	0.73
Small vs. Neutral	0.68 (0.91)	1.17 (0.98)	1.6	0.11	1.28 (0.82)	1.18 (1.24)	0.3	0.76	2.1	**0.05**	0.03	0.98

*Mean ± 1 SD of changes in ventral striatal BOLD signal contrast averaged across right and left hemispheres during anticipation of monetary incentives.*

*Reward, Trials of the monetary incentive delay task in which participants could earn or not earn money; Loss, Trials of the monetary incentive delay task in which participants could avoid losing or lose money; All incentives, All trials of the monetary incentive delay task in which an incentive was at stake.*

**FIGURE 2:**
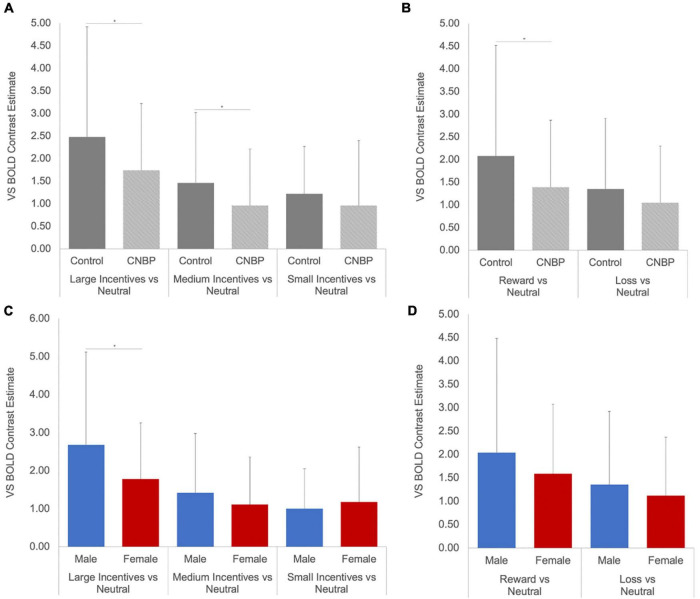
Ventral striatal responses to incentive anticipation vary by sex and group. Bars represent ventral striatal response to anticipation of each incentive broken down by sex or group then examined as a function of salience and valence. **(A)**, Salience-by-Group; **(B)**, Valence-by-Group; **(C)**, Salience-by-Sex; **(D)**, Valence-by-Sex. Error bars represent confidence intervals. VS, ventral striatum. * denotes significant differences.

A significant valence-by-group interaction [*F*_(1_,_85)_ = 5.86, *p* = 0.018] was also observed, with HC collapsed across sex exhibiting significantly greater ventral striatal activity than CNBP participants, particularly during reward trials (Figures 2A,B). These findings are consistent with those reported by Kim et al. (2020) We also obtained a significant salience-by-sex interaction [*F*_(1_,_85)_ = 15.58, *p* < 0.001; Figures 2C,D]. *Post hoc* analyses revealed males collapsed across group, relative to females collapsed across group, exhibited significantly greater BOLD signal during anticipation of large incentives (*p* = 0.02); this trend was also observed for medium and small incentives, though differences were not statistically significant. Mean ventral striatal BOLD signal comparisons between group and sex are reported in Table 3.

**TABLE 3:** Collapsed group differences in MID activity.

	Group				Sex			
	Chronic pain	Control			Male	Female		
	(*n* = 40)	(*n* = 50)			(*n* = 37)	(*n* = 53)		
	Mean (SD)	Mean (SD)	*t*	*p*	Mean (SD)	Mean (SD)	*t*	*p*
**Reward**								
All vs. Neutral	1.39 (1.02)	2.08 (1.34)	2.7	**0.008**	2.04 (1.4)	1.59 (1.2)	1.7	0.10
Large vs. Neutral	1.91 (1.37)	2.93 (2.14)	2.7	**0.008**	3.13 (2.3)	2.02 (1.4)	2.6	**0.01**
Medium vs. Neutral	1.04 (1.13)	1.60 (1.34)	2.1	**0.04**	1.52 (1.2)	1.23 (1.3)	1.1	0.29
Small vs. Neutral	1.22 (1.14)	1.73 (1.18)	2.1	**0.04**	1.46 (0.9)	1.53 (1.3)	0.29	0.78
**Loss**								
All vs. Neutral	1.05 (1.15)	1.35 (1.18)	1.2	0.23	1.36 (1.3)	1.12 (1.1)	0.97	0.33
Large vs. Neutral	1.57 (1.41)	2.03 (1.63)	1.4	0.16	2.23 (1.8)	1.55 (1.3)	2.10	**0.04**
Medium vs. Neutral	0.88 (1.33)	1.32 (1.41)	1.5	0.14	1.32 (1.6)	0.99 (1.2)	1.12	0.27
Small vs. Neutral	0.70 (1.22)	0.70 (1.39)	0.002	1.00	0.54 (1.3)	0.82 (1.3)	0.99	0.32
**All Incentives**								
All vs. Neutral	1.22 (1.03)	1.72 (1.21)	2.1	**0.04**	1.70 (1.3)	1.35 (1.0)	1.40	0.17
Large vs. Neutral	1.74 (1.28)	2.48 (1.81)	2.2	**0.03**	2.68 (2.0)	1.78 (1.2)	2.45	**0.02**
Medium vs. Neutral	0.96 (1.13)	1.46 (1.23)	2.0	**0.05**	1.42 (1.3)	1.11 (1.1)	1.21	0.23
Small vs. Neutral	0.96 (0.98)	1.22 (1.08)	1.2	0.24	1.00 (0.9)	1.18 (1.1)	0.79	0.43

*Mean ± 1 SD of changes in ventral striatal BOLD signal contrast averaged across right and left hemispheres during anticipation of monetary incentives. Reward, Trials of the monetary incentive delay task in which participants could earn or not earn money; Loss, Trials of the monetary incentive delay task in which participants could avoid losing or lose money; All incentives, All trials of the monetary incentive delay task in which an incentive was at stake.*

### Bivariate Associations

**FIGURE 3:**
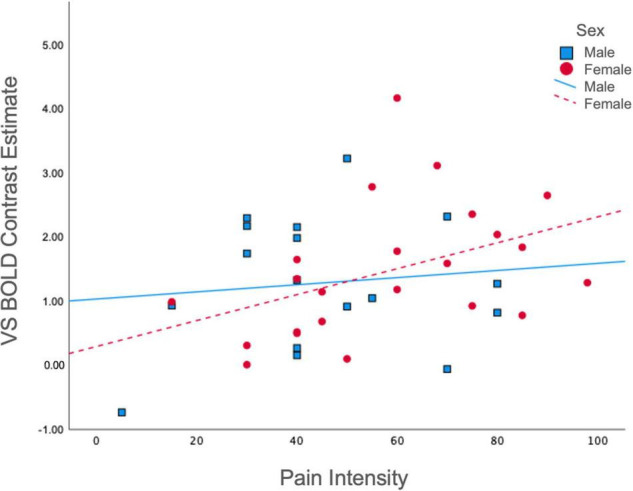
Correlations between pain intensity and ventral striatal responses during reward. Ventral Striatal BOLD during reward anticipation collapsed across all magnitudes was differentially correlated with McGill Pain Intensity scores among males (*r* = 0.12, *p* = 0.66) and females (*r* = 0.43, *p* = 0.04). VS, ventral striatum.

To gain further insight into the manner in which BOLD signal was associated with previously established clinical features pertinent to reward processing, we examined associations between average ventral striatal BOLD response during reward and loss anticipation and pain and affective measures, split between males and females. Among females, pain intensity was positively associated with BOLD in the ventral striatum during reward anticipation collapsed across all magnitudes (*r* = 0.43, *p* = 0.04); however, this was non-significant among males (*r* = 0.12, *p* = 0.66) (Figure 3). No significant correlations were obtained between pain unpleasantness, STAI, PANAS, or POMS-TMD scores and average ventral striatal BOLD signal during anticipation of reward collapsed across all magnitudes, loss collapsed across all magnitudes, or incentives collapsed across all magnitudes.

## Discussion

The present study provides the first evidence that biological sex appears to significantly impact neural responses to reward anticipation among chronic pain patients. This research builds upon and clarifies conflicting results from two previous studies examining BOLD signal among chronic pain patients and pain-free controls during a MID task (see Martucci et al., 2018; Kim et al., 2020), and highlights the importance of incorporating sex as a factor of interest in studies of reward processing among chronic pain patients.

In a sample of CNBP patients and HC, we show that ventral striatal BOLD signal was significantly lower among CNBP patients relative to HC participants during anticipation of reward—particularly during large reward trials—and that this effect is uniquely observed between patient and control males. The fact that significant findings are more pronounced during larger reward trials is perhaps unsurprising, as previous research shows ventral striatal activation during anticipation of reward corresponds to the salience and magnitude of an incentive (Cooper and Knutson, 2008). Our observed group effect is consistent with and substantiates evidence that reward processing is dysregulated in the ventral striatum among chronic back pain patients (Kim et al., 2020). Sex effects observed in the present study extend this line of research, offering unique insight into the possible mechanisms driving dysregulated reward processing among chronic pain patients.

Males and females exhibit vast differences in clinical experiences of chronic pain. Chronic pain is more prevalent among females than males overall, and within specific chronic pain syndromes, including back pain (Dahlhamer et al., 2018). In general, females endorse lower pain thresholds, lower pain tolerance, higher levels of pain intensity, and more frequently experience pain-related disability (Fillingim et al., 2009; Mogil, 2020). Our results echo this trend, as female CNBP participants reported greater pain intensity and unpleasantness than male CNBP participants. Naturally, differences in clinical manifestations of chronic pain between the biological sexes would suggest the neurobiological antecedents of these experiences are also different.

Ventral striatum alterations in reward responsivity in preclinical models yield insight into neurobiological alterations in chronic pain. Sex differences in the neural mechanisms underlying reward and associated behavior have been repeatedly observed in rodents, which is likely in part a function of gonadal hormones (Becker, 2016). For example, ovariectomized female rodents exhibit escalated motivation for and consumption of drugs of abuse following short-term estradiol treatment, as well as lesser striatal dopamine increases following cocaine intake, relative to castrated males (Becker and Koob, 2016). Furthermore, estradiol has been shown to downregulate striatal dopamine binding in ovariectomized female rats (Bazzett and Becker, 1994), and striatal dopamine release in response to amphetamine is known to be modulated by ovarian hormones, with heightened amphetamine-induced responsivity demonstrated among rats in estrus relative to those in diestrus (Becker and Cha, 1989). Similarly, behavioral differences in aversion to punishment during reward learning yield more rapid acquisition among female rodents compared with males (Chowdhury et al., 2019)—an effect necessarily preceded by neural differences.

Sex differences in reward processing among healthy humans are similarly well documented. For example, Curtis et al. (2019) observed BOLD signal in the ventral striatum was greater among males compared to females during reward trials of a gambling task. Among adolescents, Alarcón et al. (2017) have likewise shown males exhibit greater ventral striatal activity relative to females during anticipation of monetary reward in a risky-decision task. Consistent with these studies, Warthen et al. (2020) observed similar sex differences among individuals during a MID task, with males exhibiting greater BOLD activation in several regions, including the ventral striatum, in response to stimulus salience. Together, these findings implicate sexual dimorphisms in neural processing of reward, independent of chronic pain. Our findings align with these trends, showing greater striatal activity during anticipation of monetary incentives among pain-free males, relative to pain-free females.

Given that reward system dysregulation is a prominent feature of the neurobiological underpinnings of chronic pain, it is somewhat surprising that well established sex differences in reward processing seem to disappear in the context of chronic pain. Our finding that reward responsivity within the ventral striatum is altered in males while stable among females suggests (1) that chronic pain-related reward system alterations in males hinge more on ventral striatal responsivity than in females, and (2) that prototypically heightened reward responsivity to monetary incentives in males is possibly more vulnerable to disturbance by chronic pain. Meanwhile, given the positive association of ventral striatal BOLD signal with pain intensity scores uniquely observed among females, it may be that individuals who more effectively downregulate ventral striatal responses to reward anticipation are also more effectively regulating pain. Thus, it could also be the case that reward processing in females is equally vulnerable to disturbance by chronic pain, yet in contrast to males, this is due to less effectively downregulating ventral striatal activity.

Our findings in females complement those of Martucci et al. (2018) supporting a hypothesis that reward system neuroadaptations associated with chronic pain in females are largely not manifested by ventral striatal—though possibly other regional mesolimbic—responses to monetary incentives. Similarly, while our results in males echo those reported by Kim et al. (2020) it is worth noting the extent to which CNBP patients may be driving the outcome in their combined sample of low back pain patients and fibromyalgia patients. It is highly likely that ventral striatal alterations in reward processing may not be universal across all chronic pain conditions. Accordingly, future studies explicitly designed to evaluate pathophysiological differences between chronic pain conditions, and across other brain regions implicated in reward, will be important contributions for clarification of diagnostic differences—in particular with regard to differences between localized vs. widespread pain and considerations of chronic overlapping pain conditions. Additionally, the role of gonadal hormones and variability across the menstrual cycle will also be essential lines of inquiry lending further insight into our findings.

The present study provides novel evidence of sex-specific reductions in anticipatory responses to reward in patients with chronic pain. These findings hold valuable implications for future mechanistic studies of chronic pain, particularly regarding the extent to which medications commonly used to treat chronic pain (e.g., opioids) may differentially alter both neurobiological and behavioral sequelae among males vs. females. Indeed, a few studies that have evaluated brain reward processing in patients with chronic pain who take opioids show altered frontostriatal responses to reward (Martucci et al., 2019) and altered resting state frontostriatal connectivity (McConnell et al., 2020), but were not evaluated in the context of sex differences.

A few limitations should be noted in consideration of these results. In addition to the limitations imposed by our inability to account for contributions of menstrual cycle phase and gonadal hormones, our findings were derived from a relatively small sample. Moreover, our recruitment from specialized pain clinics may reduce the generalizability of results. Additionally, these results are cross-sectional. Future studies with larger, more heterogenous samples will allow for more nuanced examinations of causal effects and aid in unpacking the unique contributions of psychological, endocrine, and neurobiological variables to sex differences in reward anticipation among chronic pain patients. Menstrual cycle tracking and blood collection for hormone monitoring will also be essential additions to future studies. Finally, given the statistically significant difference in age between groups, further research is needed to explore, rather than control for, the impact of aging on reward valuation. Despite these limitations, the present study contributes innovative findings to the small existing body of literature exploring striatal hypofunction in chronic pain.

## Data Availability Statement

The raw data supporting the conclusions of this article will be made available by the authors, without undue reservation.

## Ethics Statement

The studies involving human participants were reviewed and approved by the University of Michigan Internal Review Board. The patients/participants provided their written informed consent to participate in this study.

## Author Contributions

TL and J-KZ were responsible for the study concept and design. TL wrote the study protocol and oversaw data collection. AB, TL, and LE performed statistical analyses and summarized the results. AB wrote the first draft of the manuscript. VK, BM, KM, and J-KZ assisted with interpretation of findings and provided critical revisions of subsequent manuscript drafts. All authors significantly contributed to the manuscript and approve of this submission.

## Conflict of Interest

BM has received research funding from LivaNova and Novartis and consulting fees from Alkermes, and he has served on the advisory board of FutraMed (unpaid). The remaining authors declare that the research was conducted in the absence of any commercial or financial relationships that could be construed as a potential conflict of interest.

## Publisher’s Note

All claims expressed in this article are solely those of the authors and do not necessarily represent those of their affiliated organizations, or those of the publisher, the editors and the reviewers. Any product that may be evaluated in this article, or claim that may be made by its manufacturer, is not guaranteed or endorsed by the publisher.
