# Supplementary material for: Altered Reward Processing and Sex Differences in Chronic Pain
Source: Front Neurosci. 2022 Jun 7;16:889849. doi: 10.3389/fnins.2022.889849 (PMC9211769; doi:10.3389/fnins.2022.889849)
Supplement: Supplementary file 1 [file Data_Sheet_1.PDF]

Supplementary Material

1 Supplementary Materials and Methods

1.1 fMRIPrep: Anatomical Data Pre-processing

Anatomical T1-weighted images were collected using a 3D inversion recovery-prepared fast spoiled gradient recalled sequence (3D-SPGR; Signa Excite: TR= 9.2ms, TE= 1.9ms, flip angle= 15°, 1.0mm isotropic voxels, field of view=25/26cm, 256x256 matrix; Discovery: TR= 12.2ms, TE= 5.2ms, flip angle= 15°, 1.0mm isotropic voxels, field of view=25/26cm, 256x256 matrix).

To correct for intensity non-uniformity N4BiasFieldCorrection was applied to the T1 image (1). Skull-stripping was performed using ANTs (v2.2.0) (2) and a brain mask was generated by fMRIPrep 1.3.0..post3 (3). The T1w image was normalized to the MNI152 database's ICBM 152 Nonlinear Asymmetrical template version 2009c (4) using nonlinear registration with antsRegistration (ANTs v2.2.0). Brain-extracted versions of the T1w volume and template were used during normalization.

1.2 fMRIPrep: Functional Data Pre-processing

For each of the BOLD runs, fMRIPrep created a reference BOLD image and its corresponding skull-stripped version (5). That reference image was then coregistered with the T1w reference created above using a boundary-based registration found Freesurfer (6). All head-motion parameters (x, y, z, pitch, roll, and yaw and the respective transformation matrices) were generated in reference to this BOLD reference image before any spatiotemporal filtering was applied with FSL (v5.0.9) (7). Slice timing correction was performed with 3dTshift from AFNI (v20160207) (8), and the time-series was resampled onto fsaverage5 space. Additionally, all BOLD time-series data that was resampled was put into its original, native space with one composite transform to correct for head-motion and susceptibility distortion (5). Then, the BOLD images were resampled into the same normalized space as the T1w images. ANTs used Lanczos interpolation to minimize the smoothing effects of other kernels. Non-gridded samplings were also performed using Freesurfer (5). Other internal operations depended on Nilearn 0.5.0 (9) to finish the functional pre-processing pipeline.

2 Supplementary Analysis

The use of two different scanners for data collection was a potential confound in our statistical analyses. Accordingly, we assessed whether scanner type had any effect on our results. To do this, we conducted our group by sex analyses using data collected only from the Sigma Excite scanner (n=75). We also explored whether we observed any scanner-related differences (Excite vs. Discovery) in ventral striatal BOLD signal during incentive anticipation.

3 Supplementary Results

Consistent with our combined sample findings, results from our repeated measures ANOVA using only data from the Sigma Excite showed a significant group-by-sex interaction effect ($F_{(1,70)} = 5.28$, $p = 0.025$). Post-hoc tests revealed control males, relative to control females, exhibited greater ventral striatal BOLD activation during overall reward anticipation ($p = 0.002$). Relative to control males,

patient males showed significantly less ventral striatal activation in response to overall reward anticipation ($p = 0.001$), while anticipatory activation for overall reward was similar among both patient and control females ($p = 0.75$).

Also consistent with our combined sample findings, we once again observed a significant valence-by-group interaction ($F_{(1,70)} = 5.41$, $p = 0.023$), with healthy controls exhibiting significantly greater ventral striatal activity than CNBP participants during overall reward anticipation. The previously observed significant salience-by-sex interaction was also maintained ($F_{(1,70)} = 13.51$, $p < 0.001$), with males, relative to females, exhibiting significantly greater BOLD signal during anticipation of large incentives.

In addition to testing one scanner group only, we checked for differences in ventral striatal responses to reward anticipation between scanners. Independent-samples t-tests yielded no significant differences in striatal response between scanner groups (see Table S1).

4 Supplementary Tables

Table S1. Ventral striatal responses to incentive anticipation stratified by scanner

Contrast	Signa Excite (n=75)	Discovery MR750 (n=15)	*t*	*p*
Reward				
All vs Neutral	1.83 ± 1.3	1.51 ± 1.01	0.89	0.38
Large vs Neutral	2.58 ± 1.97	1.92 ± 1.43	1.23	0.22
Medium vs Neutral	1.40 ± 1.34	1.08 ± 0.92	0.89	0.38
Small vs Neutral	1.50 ± 1.13	1.50 ± 1.49	0.10	0.92
Loss				
All vs Neutral	1.25 ± 1.17	1.04 ± 1.19	0.65	0.52
Large vs Neutral	1.91 ± 1.59	1.41 ± 1.28	1.14	0.26
Medium vs Neutral	1.22 ± 1.42	0.64 ± 1.12	1.50	0.14
Small vs Neutral	0.63 ± 1.21	1.06 ± 1.75	1.14	0.26
All Incentives				
All vs Neutral	1.54 ± 1.18	1.27 ± 1.04	0.82	0.42
Large vs Neutral	2.25 ± 1.68	1.67 ± 1.27	1.26	0.21
Medium vs Neutral	1.31 ± 1.24	0.86 ± 0.95	1.33	0.19
Small vs Neutral	1.07 ± 0.97	1.29 ± 1.36	0.78	0.44

Mean ± 1 SD of changes in ventral striatal BOLD signal contrast averaged across right and left hemispheres during anticipation of monetary incentives
